# Relationship between admission coagulopathy and prognosis in children with traumatic brain injury: a retrospective study

**DOI:** 10.1186/s13049-021-00884-4

**Published:** 2021-05-20

**Authors:** Cheng-yan You, Si-wei Lu, Yue-qiang Fu, Feng Xu

**Affiliations:** 1 Department of Critical Care Medicine, Childrens Hospital, Chongqing Medical University, 136# Zhongshan Er Road, Yu Zhong District, 400014 Chongqing, Peoples Republic of China; 2 Ministry of Education Key Laboratory of Child Development and Disorders, 400014 Chongqing, Peoples Republic of China; 3 National Clinical Research Center for Child Health and Disorders, 400014 Chongqing, Peoples Republic of China; 4 China International Science and Technology Cooperation base of Child development and Critical Disorders, 400014 Chongqing, Peoples Republic of China; 5 Chongqing Key Laboratory of Pediatrics, 400014 Chongqing, Peoples Republic of China

**Keywords:** Children, Activated partial thromboplastin time, Fibrinogen, Prognosis, Traumatic brain injury

## Abstract

**Background:**

Coagulopathy in adult patients with traumatic brain injury (TBI) is strongly associated with unfavorable outcomes. However, few reports focus on pediatric TBI-associated coagulopathy.

**Methods:**

We retrospectively identified children with Glasgow Coma Scale13 in a tertiary pediatric hospital from April 2012 to December 2019 to evaluate the impact of admission coagulopathy on their prognosis. A classification and regression tree (CART) analysis using coagulation parameters was performed to stratify the death risk among patients. The importance of these parameters was examined by multivariate logistic regression analysis.

**Results:**

A total of 281 children with moderate to severe TBI were enrolled. A receiver operating characteristic curve showed that activated partial thromboplastin time (APTT) and fibrinogen were effective predictors of in-hospital mortality. According to the CART analysis, APTT of 39.2s was identified as the best discriminator, while 120mg/dL fibrinogen was the second split in the subgroup of APTT39.2s. Patients were stratified into three groups, in which mortality was as follows: 4.5% (APTT39.2s, fibrinogen>120mg/dL), 20.5% (APTT39.2s and fibrinogen120mg/dL) and 60.8% (APTT>39.2s). Furthermore, length-of-stay in the ICU and duration of mechanical ventilation were significantly prolonged in patients with deteriorated APTT or fibrinogen values. Multiple logistic regression analysis showed that APTT>39.2s and fibrinogen120mg/dL was independently associated with mortality in children with moderate to severe TBI.

**Conclusions:**

We concluded that admission APTT>39.2s and fibrinogen120mg/dL were independently associated with mortality in children with moderate to severe TBI. Early identification and intervention of abnormal APTT and fibrinogen in pediatric TBI patients may be beneficial to their prognosis.

## Introduction

Traumatic brain injury (TBI) is one of the leading causes of trauma-induced mortality and disability in children and adolescents [1]. Coagulation dysfunction is frequently observed following TBI, especially in patients with severe TBI [2,4]. Although there is a trend toward increased use of dynamic hemostatic assays such as thromboelastography (TEG), coagulopathy is still commonly diagnosed by routine coagulation laboratory tests, including the International normalized ratio (INR), activated partial thromboplastin time (APTT) and others, in developing countries.

Current research revealed that abnormalities in routine coagulation laboratory tests upon admission were strong predictors of poor outcomes in patients with TBI [5,8]. Albert et al. [8] reported that the mortality of TBI patients with admission coagulopathy, defined by a prolonged INR, prothrombin time (PT) or APTT, was significantly higher than that of the no-coagulopathic group. Kelly et al. [6] demonstrated that D-dimer elevation upon admission was significantly associated with progression of intracranial hemorrhage (PICH), which is a major cause of secondary brain injury and a worse outcome after TBI.

However, there are relatively few reports focused on coagulation abnormalities in children with TBI. In one comparative study that included pediatric and adult trauma patients, Strumwasser et al. [9] reported that TBI-related coagulopathy increased in a stepwise fashion with age. Dwivedi et al. [3] found that coagulopathy was present in 37.5% of the pediatric TBI group and 68.03% of the adult group, and there was a high mortality rate in the coagulopathy group in both the adult and pediatric populations (63.04 and 50%, respectively). However, it is noteworthy that the population of the pediatric TBI group in their study was relatively small (*n*=48) [3]. Whittaker et al. [10] found that early coagulopathy was associated with a modest increase in mortality in pediatric trauma patients without TBI, whereas it was associated with a four-fold increase in mortality among TBI patients. Importantly, coagulopathy in this study was only defined by an INR of 1.2 or greater [10], ignoring the impact of other coagulation parameters. The impact of coagulopathy on children with TBI may be different from that of the adult population and pediatric trauma patients without TBI. Moreover, due to the heterogenic definition of coagulopathy and cutoff values used, the relationship between admission coagulopathy and clinical outcomes in children with TBI remains to be established.

In this study, we aim to evaluate the predictive value of admission coagulation parameters on the prognosis of children with moderate to severe TBI. We hypothesized that the admission coagulation parameters were associated with increased in-hospital mortality, length-of-stay in the ICU and duration of mechanical ventilation in children with moderate to severe TBI.

## Methods

### Study design and participants

 We retrospectively identified all pediatric patients younger than 18 years with moderate to severe TBI [Glasgow Coma Scale (GCS)13] who were admitted to the intensive care unit (ICU) at the Childrens Hospital of Chongqing Medical University in Chongqing, China, from April 2012 to December 2019. Ethical approval was obtained from the Institutional Ethics Review Board of Childrens Hospital, Chongqing Medical University. The need for informed consent was waived due to the anonymous and observational nature of this study. The exclusion criteria of the study were as follows: older than 18 years, injury occurred over 24h of admission, blood product transfusion prior to sample collection, history of a hemorrhagic disorder, anticoagulant medication before presentation at the Emergency Department.

### Study parameters and definitions

Blood samples were collected immediately upon arrival at the emergency department. The demographic and clinical information collected included age; gender; weight; mechanism of injury; TBI lesion type; admission GCS score; Abbreviated Injury Scale (AIS); Injury Severity Score (ISS); Pediatric Trauma Score (PTS); systolic blood pressure (SBP); routine laboratory investigations, such as lactate level, INR, APTT, thrombin time (TT), fibrinogen, platelet count (PLT), D-dimer level, white blood cell (WBC) count and hemoglobin (Hgb) level; length of stay (LOS) in the ICU and hospital; duration of mechanical ventilation; and mortality.

The Pediatric Trauma Score (PTS) was developed as a means of providing a rapid accurate assessment of the severity of injury of an injured child and includes weight, airway, SBP, central nervous system, open wound and skeletal states [11]. Hypotension was defined as an admission SBP<70 mmHg for infants younger than 1 year, SBP<70 +(2age) mmHg for patients aged from 1 to 10 years and SBP<90 mmHg for children aged 11 years and older [12]. Polytrauma was defined as AIS head3 with one other AIS region3. Abnormal pupil was defined by abnormalities in pupil size, symmetry, or pupillary light reactivity.

### Management of TBI

During hospitalization, TBI patients were given routine care for head trauma, as follows: Midazolam and phenobarbital sodium were used for sedation and sufentanil for analgesia; hyperosmolar medication (mannitol, glycerol fructose, hypertonic saline) was administered to reduce intracranial pressure when needed; temperature was controlled to avoid hyperthermia using temperature-regulating blankets; and neurosurgical interventions were performed, including monitoring of intracranial pressure, external ventricular draining, evacuation of hematoma, dissection of necrotic brain tissue, decompressive craniectomy and reduction of separation-fracture.

### Outcomes

The primary outcome measure was post-trauma mortality that occurred in the hospital. The secondary outcome measures were duration of mechanical ventilation and length of stay (LOS) in the ICU and hospital. To account for death as a competing outcome, we considered the need for mechanical ventilation as ventilator-free days with a maximum of 14 days. For LOS, we used ICU- and hospital-free days with maximums of 14 and 28 days, respectively.

### Statistical analysis

All data analyses were performed using the IBM SPSS Statistics for Windows, version 23.0 (IBM Corp, Armonk NY, USA). Receiver operating characteristic (ROC) curves were calculated using Medcalc (version 19.0.7 Ostend, Belgium). Baseline characteristics were compared between survivors and non-survivors during the hospital stay. Continuous variables are expressed as the median and interquartile range (IQR) and were compared using the Mann Whitney U-test for two groups or the Kruskal-Wallis H test for more than two groups. Categorical variables are expressed as percentages, and the Chi-Square test/Fishers test was performed to compare proportions. A *p* value of <0.05 was considered statistically significant.

ROC curves were used to assess the predictive performance of routine coagulation parameters for mortality in the overall TBI group and the subgroup of isolated TBI. The classification and regression tree (CART) is a predictive model used to explore variables that exist as multicollinearity problems by selecting only one variable as the most important discriminator, and it has become increasingly popular in the medical field [13]. Considering this, we performed a CART analysis to investigate which coagulation parameters best predicted mortality risk and their optimal cut-off values. Finally, based on the cut-off value from the CART analysis, multivariate logistic regression analysis was performed to identify risk factors independently associated with mortality after adjusting for other factors that were significant at *p*<0.05.

## Results

### Clinical characteristics

A total of 281 children with moderate to severe TBI were enrolled. Of these children, 53 (18.9%) died in the hospital. The baseline and clinical characteristics of the survivors (*n*=228) and non-survivors (*n*=53) in the overall group and the isolated TBI group are summarized respectively in Tables1 and 2. Males comprised 60.5% of the total study population. Survivors had significantly a higher GCS and PTS than non-survivors (*p*<0.05) in both the overall group and isolated TBI group, which indicated that the injury severity was closely associated with death. However, the comparison of ISS between survivors and non-survivors in the isolated TBI group was of no significance, which may due to the relative mild damage to other parts of the body in the isolated TBI group. Fall was the leading cause of TBI in both the overall group and the isolated TBI group (135, 48.0% and 63, 46.0% respectively), the rest of the causes of the TBI cases were road traffic accidents (109, 38.8% and 43, 31.4% respectively) and others. The most common intracranial lesions in the enrolled patients in decreasing order of frequency were as follows: subarachnoid hemorrhage (137, 48.8%), contusion (125, 44.5%), subdural hemorrhage (98, 34.9%), extradural hemorrhage (82, 29.2%) and intraparenchymal hemorrhage (42, 14.9%).

**Table 1:** Demographics and clinical data of all TBI patients by survivors and non-survivors

	All patients (*n*=281)	Survivors (*n*=228)	Non-survivors (*n*=53)	*P* value
Baseline Demographics				
Age, M	50.0 (19.587.0)	51.0 (20.387.0)	49.0 (18.0-85.5)	0.678
Weight, kg	17.0 (11.324.0)	17.5 (11.025.0)	15.0 (12.0-21.3)	0.419
Gender, male, n (%)	170 (60.5%)	141 (61.8%)	29 (54.7%)	0.339
Injury severity				
GCS	8 (610)	8 (711)	5 (37)	<0.001
PTS	5 (37)	5 (37)	2 (14)	<0.001
ISS	18 (1327)	18 (925)	25 (1830)	0.003
Mechanism of injury				0.505
Traffic, n (%)	109 (38.8%)	92 (40.4%)	17 (32.1%)	
Fall, n (%)	135 (48.0%)	106 (46.5%)	29 (54.7%)	
Others, n (%)	37 (13.2%)	30 (13.2%)	7 (13.2%)	
Lesion type				
EDH, n (%)	82 (29.2%)	74 (32.5%)	8 (15.1%)	0.018
SDH, n (%)	98 (34.9%)	78 (34.2%)	20 (37.7%)	0.476
SAH, n (%)	137 (48.8%)	106 (46.5%)	31 (58.5%)	0.057
Contusion, n (%)	125 (44.5%)	102 (44.7%)	23 (43.4%)	0.932
Intraparenchymal hemorrhage,n(%)	42 (14.9%)	36 (15.8%)	6 (11.3%)	0.477
Polytrauma, n (%)	144 (51.2%)	110 (48.2%)	34 (64.2%)	0.037
Abnormal pupil, n (%)	109 (38.8%)	62 (27.2%)	47 (88.7%)	<0.001
Hypotension, n (%)	34 (12.1%)	19 (8.3%)	15 (28.3%)	<0.001
Laboratory investigations				
Lactate, mmol/L	2.1 (0.8-4.0)	1.5 (0.83.2)	4.6 (2.9-7.0)	<0.001
INR	1.16 (1.071.34)	1.14 (1.051.26)	1.49 (1.211.94)	<0.001
APTT, s	29.4 (26.0-35.3)	28.6 (25.432.5)	44.4 (29.668.3)	<0.001
Fibrinogen, mg/dL	134.0 (82.0-182.0)	143.0 (104.8188.0)	71.0 (50.0-112.0)	<0.001
TT, s	18.7 (17.321.3)	18.3 (17.120.3)	22 (18.830.1)	<0.001
D-dimer, ug/L	14,040 (396522,865)	13,665 (379022,348)	16,460 (528029,695)	0.127
PLT, /uL	261.0 (200.0-324.0)	264.5 (208.3-331.3)	210.0 (158.0-298.0)	0.006
Hgb, g/L	98.0 (81.5111.0)	101.0 (84.3113.0)	85.0 (68.0-102.0)	<0.001
WBC, *10^9/L	16.6(12.221.5)	16.7(12.221.1)	15.9(12.723.1)	0.486

Data are shown as the median (interquartile range) or a number (percentage)

*M*month, *GCS*Glasgow Coma Score, *PTS*Pediatric Trauma Score, *ISS*Injury Severity Score, *EDH*epidural hemorrhage, *SDH*subdural hemorrhage, *SAH*subarachnoid hemorrhage, *INR*International normalized ratio, *APTT*activated partial thromboplastin time, *TT*Thrombin time, *PLT*platelet count, *HgB*hemoglobin, *WBC*white blood cell

**Table 2:** Demographics and clinical data of isolated TBI patients by survivors and non-survivors

	Isolated TBI patients (*n*=137)	Survivors (*n*=118)	Non-survivors (*n*=19)		*P* value	
Baseline Demographics						
Age, M	36.0 (12.579.0)	45.0 (13.080.0)	17.0(11.058.0)		0.112	
Weight, kg	15.0 (10.022.0)	15.5 (10.023.0)	12.0 (9.015.0)		0.117	
Gender, male, n (%)	78 (56.9%)	69 (58.5%)	9 (47.4%)		0.456	
Injury severity						
GCS	9 (712)	9 (712)	6 (48)		0.001	
PTS	6 (48)	7 (48)	4 (26)		0.001	
ISS	16 (918)	16 (916)	16 (925)		0.119	
Mechanism of injury					0.668	
Traffic, n (%)	43 (31.4%)	39 (33.1%)	4 (21.1%)			
Fall, n (%)	63 (46.0%)	54 (45.8%)	9 (47.4%)			
Others, n (%)	31 (22.6%)	25 (21.2%)	6 (31.6%)			
Abnormal pupil, n (%)	44 (32.1%)	28 (23.7%)	16 (84.2%)		<0.001	
Hypotension, n (%)	12 (8.8%)	9 (7.6%)	3 (15.8%)		0.373	
Laboratory investigations						
Lactate, mmol/L	1.4 (0.83.4)	1.2 (0.72.7)	4.4 (2.88.3)		<0.001	
INR	1.12 (1.031.24)	1.11 (1.031.20)	1.22 (1.091.49)		0.027	
APTT, s	28.6 (25.0-33.2)	27.7 (24.732.7)	29.7 (27.547.4)		0.034	
Fibrinogen, mg/dL	143.0 (97.0-190.0)	150.0 (110.0-194.0)	71.0 (105.0-157.0)		0.024	
TT, s	18.4 (17.321.2)	18.3 (17.120.7)	18.9 (17.922.1)		0.082	
D-dimer, ug/L	9810 (238517,865)	10,430 (241318,458)	5540 (157016,600)		0.645	
PLT, /uL	283.0 (214.5-344.5)	283.0 (216.5343.0)	262.0 (203.0-420.0)		0.830	
Hgb, g/L	101.0 (81.5-112.5)	103.0 (81.8-114.3)	90.0 (78.0-102.0)		0.044	
WBC, *10^9/L	15.7 (11.921.1)	15.7 (11.921.0)	14.3 (11.421.7)		0.746	

Data are shown as the median (interquartile range) or a number (percentage)

*M*month, *GCS*Glasgow Coma Score, *PTS*Pediatric Trauma Score, *INR*International normalized ratio, *APTT*activated partial thromboplastin time, *TT*Thrombin time, *PLT*platelet count, *Hgb*hemoglobin, *WBC*white blood cell

One hundred forty-four (51.2%) patients presented with polytrauma. Compared with survivors, non-survivors showed significantly higher rates of hypotension and abnormal pupil at admission in the overall group (28.3% vs. 8.3%, 88.7% vs. 27.2%). While in the subgroup of isolated TBI patients, the incidence of admission hypotension in non-survivors and survivors was of no significance (15.8% vs. 7.6%, *p*=0.373). The baseline admission lactate level, INR and APTT were significantly higher in non-survivors than survivors in both the overall group and the subgroup of isolated TBI patients, while the reverse was true of the fibrinogen level and Hgb concentration. The platelet count was significant decrease in the overall group (*p*=0.006), but this change was not observed in the subgroup of isolated TBI patients (*p*=0.830). However, the D-dimer level and WBC count were not significantly elevated in non-survivors compared to survivors in both the overall group and the isolated TBI group.

According to the comparisons of demographics and clinical data in patients with isolated TBI and polytrauma in Table3, we found that the age, weight, and severity of injury in polytrauma patients were significantly greater than isolated TBI patients, and the incidence of traffic and fall was greater than patients in isolated TBI group. This may due to the relatively wider range of activities among elder children. Compared with the isolated TBI patients, the percentage of abnormal pupil, the level of admission lactate, INR, APTT, D-dimer were significantly elevated in children with polytrauma, and the level of fibrinogen and platelet was significantly decreased in polytrauma patients. In addition, we found that the rate of hypotension, TT and Hgb concentration were of no significance in the two groups.

**Table 3:** Comparison between isolated TBI patients and polytrauma patients

	Isolated TBI (*n*=137)			TBI+other (*n*=144)		*P* value
Baseline Demographics						
Age, M	36.0 (12.579.0)			60.5 (29.396.0)		<0.001
Weight, kg	15.0 (10.022.0)			19.5 (13.625.0)		0.001
Gender, male, n (%)	78 (56.9%)			92 (63.9%)		0.233
Injury severity						
GCS	9 (712)			7 (58)		<0.001
PTS	6 (48)			4 (26)		<0.001
ISS	16 (9-17.5)			25 (1834)		<0.001
Mechanism of injury						<0.001
Traffic, n (%)	43 (31.4%)			66 (45.8%)		
Fall, n (%)	63 (46.0%)			72 (50.0%)		
Others, n (%)	31 (22.6%)			6 (4.2%)		
Abnormal pupil	44 (32.1%)			65 (45.1%)		0.028
Hypotension, n (%)	12 (8.8%)			22 (15.3%)		0.103
Laboratory investigations						
Lactate, mmol/L	1.4 (0.83.4)			2.8 (1.14.4)		0.001
INR	1.12 (1.031.24)			1.24 (1.121.51)		<0.001
APTT, s	28.6 (25.0-33.2)			30.4 (27.439.2)		0.001
Fibrinogen, mg/dL	143.0 (97.0-190.0)			123.0 (72.0-178.0)		0.033
TT, s	18.4 (17.321.2)			18.9 (17.321.8)		0.26
D-dimer, ug/L	9810 (238517,865)			16,775 (709828,523)		<0.001
PLT, /uL	283.0 (214.5-344.5)			247.5 (167.8-299.5)		0.001
Hgb, g/L	101.0 (81.5-112.5)			98.0 (81.3110.0)		0.283
WBC, *10^9/L	15.7(11.921.1)			13.1(12.722.0)		0.120

Data are shown as the median (interquartile range) or a number (percentage)

*M*month, *GCS*Glasgow Coma Score, *PTS*Pediatric Trauma Score, *ISS*Injury Severity Score, *INR*International normalized ratio, *APTT*activated partial thromboplastin time, *TT*Thrombin time, *PLT*platelet count, *Hgb*hemoglobin, *WBC*white blood cell

### ROC curve analyses of in-hospital mortality

ROC curve analysis in the overall group (Table4) showed that the INR (AUC=0.770, 95% CI: 0.7160.818, *p*<0.001), APTT (AUC=0.781, 95% CI:0.7280.828, *p*<0.001), TT (AUC=0.749, 95% CI:0.6940.798, *p*<0.001), fibrinogen level (AUC=0.759, 95% CI: 0.7040.808, *p*<0.001), and PLT (AUC=0.621, 95% CI:0.5620.678, *p*=0.01) were effective predictors of in-hospital mortality in children with moderate to severe TBI. We also performed ROC curve analysis of conventional coagulation tests for in-hospital mortality in the subgroup of isolated TBI patients (Table5). The results showed that admission INR (AUC 0.659, 95%CI 0.4970.821, *P*=0.027), APTT (AUC 0.652, 95%CI 0.5150.789, *P*=0.034), fibrinogen (AUC 0.662, 95%CI 0.5190.805, *P*=0.024) could also effectively predict the in-hospital mortality. But the area under the curve was not as large as in the overall group.

**Table 4:** ROC curve analysis of conventional coagulation tests for in-hospital mortality in all TBI patients

Variables	AUC	95%CI	*P*
INR	0.770	0.7160.818	<0.001
APTT	0.781	0.7280.828	<0.001
TT	0.749	0.6940.798	<0.001
Fibrinogen	0.759	0.7040.808	<0.001
PLT	0.621	0.5620.678	0.01

*AUC*area under the curve, *CI*confidence interval, *INR*International normalized ratio, *APTT*activated partial thromboplastin time, *TT*Thrombin time, *PLT*platelet

**Table 5:** ROC curve analysis of conventional coagulation tests for in-hospital mortality in isolated TBI patients

Variables	AUC	95%CI	*P*
INR	0.659	0.4970.821	0.027
APTT	0.652	0.5150.789	0.034
TT	0.624	0.4890.759	0.082
Fibrinogen	0.662	0.5190.805	0.024
PLT	0.485	0.3270.642	0.830

*AUC*area under the curve, *CI*confidence interval, *INR*International normalized ratio, *APTT*activated partial thromboplastin time, *TT*Thrombin time, *PLT*platelet

### CART analysis for in-hospital mortality

**Fig. 1:**
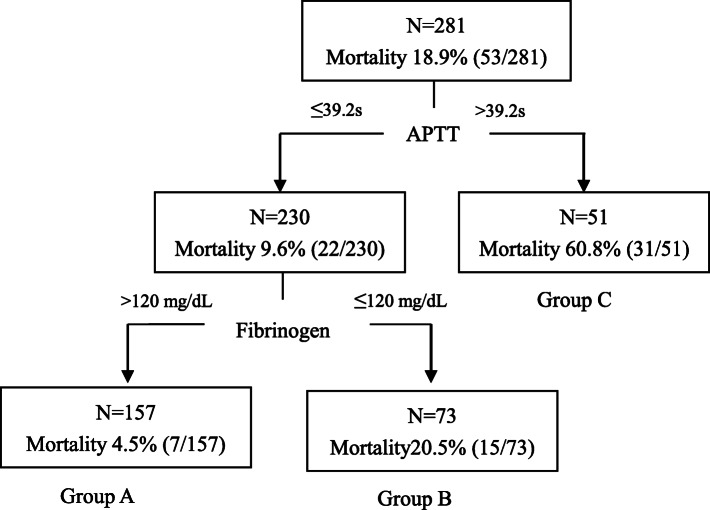
CART prediction model for mortality in children with moderate to severe TBI.The selected splitting variables (APTT of 39.2s and fibrinogen of 120mg/dL) are shown in the nodes. Three groups were established: group A (APTT39.2s, fibrinogen>120mg/dL, *n*=157), group B (APTT39.2s, fibrinogen120mg/dL, *n*=73) and group C (APTT>39.2s, *n*=51). APTT, activated partial thromboplastin time

CART analysis was carried out using coagulation parameters, including the INR, APTT, TT, fibrinogen, and PLT, to predict mortality in children with TBI (Fig.1). Consequently, APTT was identified as the best discriminator, with an optimal cut-off value of 39.2s; 120mg/dL fibrinogen was the second split in the subgroup of APTT39.2s. As a result, patients were stratified into three groups: a low-risk group (group A) with APTT39.2s and fibrinogen>120mg/dL, an intermediate-risk group (group B) with APTT39.2s and fibrinogen120mg/dL, and a high- risk group (group C) with APTT>39.2s. The mortality rates for groups A, B and C were 4.5%, 20.5 and 60.8%, respectively. The predictive accuracy of the CART model was 85.1%, and the risk of misclassification was 18.9%.

### Clinical characteristics of Group A, Group B and Group C established by CART

The clinical characteristics of group A, group B and group C are compared in Table6. Compared with group A and B, children in group C had significantly more severe injury scores. The proportions of polytrauma and hypotension in group C were significantly higher than those in group A and B, respectively. Significantly elevated lactate and decreased Hgb were also observed in group C compared to groups A and B. Increased in-hospital mortality and prolonged durations of mechanical ventilation and ICU stay were observed in groups B and C compared to group A. Additionally, decreased ventilator-free days, ICU-free days and hospital-free days were observed in groups B and C compared to group A. However, the LOS in hospital in group C was less than in groups A and B, which may due to the higher mortality rate of group C.

**Table 6:** Comparison of clinical characteristics among groups A, B and C

	Group A (*n*=157)	Group B (*n*=73)	Group C (*n*=51)	*P*^a^	Pairwise Comparisons		
					A v B	A v C	B v C
Age, M	60.0 (23.0-95.5)	36.0 (14.588.0)	37.0 (21.078.0)	0.056			
Males, n (%)	90 (57.3%)	48 (65.8%)	32 (62.7%)	0.446			
Weight, kg	18.0 (12.325.0)	16.0 (10.522.0)	15.0 (10.020.0)	0.073			
GCS	8 (712)	8 (69)	5 (37)	<0.001	0.038	<0.001	<0.001
PTS	6 (48)	4 (36)	2 (13)	<0.001	0.003	<0.001	<0.001
ISS	18 (925)	18 (1229)	25 (1834)	<0.001	0.333	<0.001	0.067
Polytrauma,n (%)	72 (45.9%)	37 (50.7%)	35 (68.6%)	0.018			
Hypotension,n(%)	6 (3.8%)	10 (13.7%)	18 (35.5%)	<0.001			
Lactate, mmol/L	1.3 (0.72.8)	2.8 (1.14.4)	4.9 (2.97.1)	<0.001	<0.001	<0.001	0.001
Hgb, g/L	106 (92118)	92 (79105)	76 (5585)	<0.001	<0.001	<0.001	0.001
Death, n (%)	7 (4.5%)	15 (20.5%)	31 (60.8%)	<0.001			
HLOS, d	20 (1237)	20 (1234)	6 (125)	<0.001	1	<0.001	0.001
Hospital-free days, d	7 (015)	2 (012)	0 (00)	<0.001	0.148	<0.001	<0.001
ILOS, d	2 (15)	4 (111)	4 (19)	0.01	0.017	0.146	1
ICU-free days, d	12 (913)	7 (013)	0 (01)	<0.001	<0.001	<0.001	<0.001
Intubation days, d	1 (12)	2 (16)	2 (16)	<0.001	<0.001	0.001	1
Intubation-free days, d	13 (1113)	6 (010)	0 (00)	<0.001	<0.001	<0.001	<0.001

Data are shown as the median (interquartile range) or a number (percentage)

*M*month, *GCS*Glasgow Coma Score, *PTS*Pediatric Trauma Score, *ISS*Injury Severity Score, *Hgb*hemoglobin, *HLOS*hospital length-of-stay, *ICU*intensive care unit, *ILOS*ICU length-of-stay

^a^KruskalWallis or Chi-square test for continuous or categorical variables, respectively

### Multivariate logistic regression analysis for mortality

Based on the cut-off value from the CART analysis, multivariate logistic regression analysis was performed to assess the effect of abnormal coagulation parameters, including APTT>39.2s, fibrinogen120mg/dL, hypotension, GCS8, ISS16, Hgb<90g/L, lactate>2 mmol/L, polytrauma and abnormal pupil. Consequently, we concluded that APTT>39.2s (OR=13.189, 95% CI4.101-42.411, *P*<0.001), fibrinogen120mg/dL (OR=3.871, 95% CI1.443-10.388, *P*=0.007), and abnormal pupil (OR=16.680, 95% CI5.405-51.474, *P*<0.001) were independently associated with in-hospital mortality in the overall TBI patients (Table7).

**Table 7:** Multivariate logistic regression analysis in all TBI patients to identify risk factors at admission related to mortality

Variate	Univariate logistic regression unadjusted OR (95% CI)	*P* value	Multivariate logistic regression adjusted OR (95% CI)	*P* value
APTT>39.2s	14.655 (7.180-29.911)	<0.001	13.189 (4.10142.411)	<0.001
Fibrinogen120mg/dL	8.107 (3.94716.652)	<0.001	3.871 (1.44310.388)	0.007
Hypotension	4.342 (2.0309.286)	<0.001	0.783 (0.2322.642)	0.694
GCS8	5.905 (2.42714.369)	<0.001	1.130 (0.3443.707)	0.840
ISS16	2.391 (1.0705.341)	0.034	0.640 (0.1952.103)	0.462
Lactate>2 mmol/l	13.544 (5.190-35.344)	<0.001	2.657 (0.8368.444)	0.098
Hgb<90g/L	3.116 (1.6865.758)	<0.001	0.515 (0.1811.463)	0.213
Abnormal pupil	20.973 (8.54151.502)	<0.001	16.680 (5.40551.474)	<0.001
Polytrauma	1.920 (1.0343.563)	0.039	0.865 (0.3282.281)	0.769

*OR*odds ratio, *CI*confidence interval, *APTT*activated partial thromboplastin time, *GCS*Glasgow Coma Score, *ISS*Injury Severity Score, *Hgb*hemoglobin

## Discussion

Coagulation disorders after TBI can refer to the disruption of the balance between coagulation and anticoagulation after injury [14]. The proposed mechanisms that trigger hemostatic disorders after TBI are still complex and controversial. The most widely accepted hypothesis is the activation of tissue factors, which are released from the damaged brain and lead to the depletion of coagulation factors [15]. Endothelial activation, protein C pathway activation, and endogenous plasminogen activator release were also found to contribute to the occurrence of coagulopathy in TBI patients [16,18].

In our analysis, children with APTT>39.2s or fibrinogen120mg/dL were more likely to present with lower GCS and PTS and higher ISS, consistent with other studies on patients with TBI [5, 19]. Joseph et al. [19] defined coagulopathy as a prolonged INR and APTT or a decreased platelet count and found that adult TBI patients with coagulopathy were more likely to present with a lower GCS score and higher ISS. Talving et al. [20] identified GCS8 and ISS16 as independent risk factors for the development of coagulopathy in adult TBI patients. However, to the best of our knowledge, there is limited research that has explored the relationship between PTS and coagulopathy presentation. According to our study, a lower PTS was also associated with deteriorated APTT or fibrinogen values, similar to GCS and ISS.

We found that the occurrence of hypotension was significantly higher in TBI children with deteriorated APTT or fibrinogen values. Serum lactate is a by-product of anaerobic metabolism and is always used as a marker of tissue hypoperfusion. In a previous study, we demonstrated that admission serum lactate could effectively predict mortality in children with moderate to severe TBI [21]. Moreover, in the present study, serum lactate was greatly increased in patients with abnormal APTT or fibrinogen. Lactate may be closely related to coagulation dysfunction in patients with TBI. Simone et al. [22] demonstrated that TBI patients with acute coagulopathy had a higher lactate level than patients without coagulopathy and that increased lactate levels significantly correlated with decreased fibrinogen levels and increased D-dimers. However, Hayakawa et al. [7] found that the increase in lactate level was correlated with the impairment of coagulation and fibrinolytic parameters in the non-TBI group only; no such correlation was observed in the TBI group. Therefore, in children with traumatic brain injury, the relationship between the early lactate level and coagulation dysfunction requires further study.

APTT can sensitively reflect the consumption and/or dysfunction of factors XI, IX, and VIII [23]. APTT is commonly used to define coagulation dysfunction and is significantly associated with a poor clinical outcome in TBI patients [2, 5]. Nakae et al. [24] found that upward trends of APTT on admission and 3h after injury were significant negative prognostic indicators in TBI patients, and multivariate logistic regression analysis identified that APTT>30.2s was an independent risk factor for poor prognosis. In our analysis, ROC curve analysis showed that admission APTT can effectively predict mortality in children with moderate to severe TBI, and according to CART analysis, APTT was the optimal coagulation parameter to predict mortality and APTT>39.2s was independently associated with mortality after adjusting to other potential confounding factors. We argue that the APTT value at admission may potentially be useful as a predictor of mortality, which may help clinicians to better assess the severity of injury and take specific interventions to improve the prognosis of children with moderate to severe TBI.

Decreased fibrinogen is associated with deleterious effects for TBI. Tareq et al. [25] found that TBI patients in the PICH group showed a higher rate of decreased fibrinogen (fibrinogen level<2g/L) than patients in the non-PICH group. When the fibrinogen level was <2g/L at arrival, a PICH progression was three-times more likely to occur. Nakae et al. [26] revealed that among patients with TBI who did not receive a fresh frozen plasma transfusion, a fibrinogen level<150mg/dL at 3h after injury was significantly associated with a higher duration of mechanical ventilation and lower GOS scores at discharge and 3 months after injury. From the CART analysis in the present study, we concluded that TBI patients in group B with normal APTT and decreased fibrinogen (120mg/dL) had increased in-hospital mortality, prolonged LOS in the ICU and hospital, and shortened ventilator-free days, ICU-free days, and hospital-free days than patients in group A, which indicated that fibrinogen was also an important coagulation parameter associated with poor outcome.

In addition to APTT and fibrinogen, other coagulation parameters were also found to significantly associate with mortality in TBI patients. Christian et al. [27] found that radiologic and clinical deterioration of TBI patients was significantly associated with an elevated INR. Joseph et al. [19] reported that a platelet count of 10010^3^/uL or less was the strongest predictor of progression of the initial insult in repeat head computed tomography, need for neurosurgical intervention, and mortality in TBI patients. In the present study, we found that the platelet counts in the survivors and non-survivors were in the normal range, but the median platelet count was significantly lower in the non-survivor group, but this change was not observed in the subgroup of isolated TBI patients. We also observed that INR was significantly elevated in non-survivors compared to survivors and ROC curve analysis showed that INR could effectively predict mortality in children with TBI.

There were several limitations in our study. First, this is a single-center retrospective study. Since the sample size of isolated TBI patients in our study is relatively small, the stability of CART is limited. We only performed CART analysis and multivariate logistic regression analysis in the overall group and further multicenter researches is needed to explore the predictive value of admission coagulopathy for mortality in children with isolated TBI. Second, we did not evaluate the impact of the treatment of coagulation disorders on the outcome during hospitalization, such as the use of fresh frozen plasma, cryoprecipitate or platelets. Third, we only studied the impact of blood biomarkers collected at the time of admission on the prognosis of patients. The coagulation parameters at other time points after admission were not explored. Finally, we regard in-hospital mortality as the primary outcome, but longer-term functional outcomes were not evaluated.

## Conclusions

Based on our study, the presence of admission of APTT>39.2s and fibrinogen120mg/dL were independently associated with increased in-hospital mortality in pediatric TBI patients. Early identification and intervention of abnormal APTT and fibrinogen in pediatric TBI patients may be beneficial to their prognosis.

## Data Availability

Anonymized data analyzed for the current study will be shared if a reasonable request is made by a qualified investigator to the corresponding author.
